# Sequence Variability and Geographic Distribution of Lassa Virus, Sierra Leone

**DOI:** 10.3201/eid2104.141469

**Published:** 2015-04

**Authors:** Tomasz A. Leski, Michael G. Stockelman, Lina M. Moses, Matthew Park, David A. Stenger, Rashid Ansumana, Daniel G. Bausch, Baochuan Lin

**Affiliations:** Naval Research Laboratory, Washington, DC, USA (T.A. Leski, M.G. Stockelman, D.A. Stenger, B. Lin);; Tulane University, New Orleans, Louisiana, USA (L.M. Moses);; Tulane School of Public Health and Tropical Medicine, New Orleans (L.M. Moses, D.G Bausch), Thomas Jefferson High School, Alexandria, Virginia, USA (M. Park);; Mercy Hospital Research Laboratory, Bo, Sierra Leone (R. Ansumana);; Liverpool School of Tropical Medicine, Liverpool, UK (R. Ansumana);; Njala University, Bo (R. Ansumana)

**Keywords:** Lassa virus, Sierra Leone, Mastomys natalensis, multimammate rats, sequence diversity, viruses, zoonoses

## Abstract

Lassa virus (LASV) is endemic to parts of West Africa and causes highly fatal hemorrhagic fever. The multimammate rat (*Mastomys natalensis*) is the only known reservoir of LASV. Most human infections result from zoonotic transmission. The very diverse LASV genome has 4 major lineages associated with different geographic locations. We used reverse transcription PCR and resequencing microarrays to detect LASV in 41 of 214 samples from rodents captured at 8 locations in Sierra Leone. Phylogenetic analysis of partial sequences of nucleoprotein (NP), glycoprotein precursor (GPC), and polymerase (L) genes showed 5 separate clades within lineage IV of LASV in this country. The sequence diversity was higher than previously observed; mean diversity was 7.01% for nucleoprotein gene at the nucleotide level. These results may have major implications for designing diagnostic tests and therapeutic agents for LASV infections in Sierra Leone.

## Abstract 2

Circulating strains cluster geographically and belong to at least 5 distinct clades.

Lassa fever (LF) belongs to a group of viral hemorrhagic fevers characterized by a febrile syndrome and high case-fatality rates (*1*). LF differs from most viral hemorrhagic fevers in that it is endemic to a large geographic area of sub-Saharan Africa. Human cases of LF have been reported in (or imported from) Guinea, Sierra Leone, Liberia, Mali, Burkina Faso, and Nigeria; however, LF outbreaks seem to be restricted to Guinea, Sierra Leone, Liberia (the Mano River Union region), and Nigeria (*2*–*4*). In some areas of Sierra Leone and Guinea, more than half of the population has antibodies against Lassa virus (LASV; family *Arenaviridae*), the etiologic agent of LF (*5*,*6*). According to various estimates, 300,000–500,000 cases of LF result in 5,000–10,000 deaths annually in West Africa (*6*,*7*). An analysis based on seroepidemiologic data suggested that the number of cases might be much higher, reaching 3 million cases and 67,000 fatalities per year (*8*). Overall, the population at risk might include as many as 200 million persons living in a large swath of West Africa from Senegal to Nigeria and beyond (*4*).

LASV can cause infection in the multimammate rat (*Mastomys natalensis*), a natural host and reservoir of this pathogen (*9*,*10*). The multimammate rat is a commensal rodent ubiquitous in Africa (*11*,*12*). Although the routes of LASV infection are poorly characterized, humans probably get infected by eating contaminated food (*13*), by inhaling virus-contaminated aerosols (*14*), or while butchering infected rat meat (*15*). Person-to-person transmission of LASV is well documented, mostly in the form of nosocomial outbreaks (*13*).

Like other arenaviruses, LASV is an enveloped virus with a bisegmented single-stranded RNA genome encoding 4 proteins using an ambisense coding strategy (*16*). The small segment contains genes for the glycoprotein precursor (GPC) and nucleoprotein (NP), which serves as the main viral capsid protein. The large segment encodes the small zinc-binding protein (Z), which contains a RING motif, and another gene (L) containing the RNA-dependent RNA polymerase domain.

Complete genome sequences are available for several LASV strains, as are a considerable number of partial sequences from isolates originating from humans and rodents (*17*–*20*). Their analysis revealed the existence of high sequence diversity (up to 27% nt) and 4 major lineages of LASV, which correlate with geographic location (*17*). Lineages I, II, and III, and the greatest diversity of LASV strains, were found among isolates from Nigeria, whereas strains from Guinea, Sierra Leone, and Liberia seemed to be more closely related and belong exclusively to lineage IV. Sequence of the AV strain (*21*) and recently published sequences from rodent LASV isolates from Mali (*18*) suggest the existence of an additional clade (proposed as lineage V) (*22*). LASV sequences of isolates from humans and rodents are found interspersed throughout the phylogenetic tree, which is consistent with the notion that human cases typically result from transmission from rodents (*17*).

The high degree of sequence divergence of LASV genomes is a major problem affecting the development of molecular and immune-based diagnostic technologies, vaccines, and possibly antiviral drugs (*13*,*16*,*17*,*23*–*25*). Forty-seven unique partial LASV sequences from Sierra Leone were available in GenBank at the time of this analysis, which included fragments of NP (27 sequences), GPC (9 sequences), L (9 sequences), and Z (2 sequences) genes plus full sequences of small and large segments of 2 strains—Josiah and NL. Most of these sequences are from isolates collected >30 years ago; only 2 more recent sequences (GPC and L gene fragments) from strain SL06-2057 were isolated in 2006 (*17*,*19*). 

To fill this gap, we investigated the sequence diversity of strains circulating among small rodents captured in peridomestic settings in Sierra Leone. In 2014, we screened 214 samples collected during 2009 from several species of rodents trapped in villages where LF was reported in humans. We used diagnostic reverse transcription PCR (RT-PCR) and high-density resequencing microarrays to detect LASV and amplify fragments of NP, GPC, and L genes. The obtained amplicons were sequenced and compared with previously published sequences from Sierra Leone to obtain a more complete and updated picture of the strains circulating in this country.

## Methods

### Rodent Sample Collection

The rodent samples collected were part of a separate project (L.M. Moses, unpub. data). Thirteen locations were selected for study in the LF-endemic region of eastern Sierra Leone. The geographic coordinates of the sampling locations and details of rodent trapping methods are available in the online Technical Appendix (Technical Appendix Table 1). Traps with captured small animals were processed in remote areas outside of the villages according to approved guidelines (*26*). The animals were anesthetized with isoflurane, and their morphometrics recorded. Animals were euthanized by exsanguination using cardiac puncture or cervical dislocation, and necropsies were performed. Spleen sections were stored in RNALater or TRIzol for RNA extraction (Life Technologies, Grand Island, NY, USA). Rodents were identified to the genus level in the field. Animals identified as *Mastomys* sp. were further identified down to species level by using molecular methods as described previously (*27*).

**Table 1:** PCR and sequencing primers used in study of Lassa virus, Sierra Leone*

Primer name	Sequence, 5′ → 3′	Target gene	Amplicon size	Reference
1010C	TCIGGIGAIGGITGGCC	NP	670	(*17*)
OW1696R	AIATGAIGCAGTCCAIIAGTGCACAGTG			(17)
LAS_NP_F_1	GGGTGGCCATAYATTGCATC		650	This study
LAS_NP_R_1	GTCCAGGAGTGCACAGTGAG			This study
36E2	ACCGGGGATCCTAGGCATTT	GPC	317	(*24*)
LVS339-rev	GTTCTTTGTGCAGGAMAGGGGCATKGTCAT			(*24*)
LVL3359-F	AGAATYAGTGAAAGGGARAGCAATTC	L	394	(*28*)
LVL3754-R	CACATCATTGGTCCCCATTTACTRTGATC			(*28*)

*GPC, glycoprotein precursor; L, polymerase; NP, nucleoprotein.

### Nucleic Acid Extraction

RNA from 10 mg of spleen of each rodent was extracted with TRIzol following the manufacturer’s recommendations. The samples were stored at –80°C.

### RT-PCR and Sequencing

RNA were reverse-transcribed by using the SuperScript III Reverse Transcriptase kit (Life Technologies) according to the manufacturer’s instructions, and RT products were stored at –20°C. Specific oligonucleotide primer pairs were used for the PCR targets of interest (Table 1) at final concentrations of 0.25 μM each. For PCR, 2 μL of RT reaction was used as template in 25 μL reactions containing 1.25 mM dNTPs, 1× Taq buffer, 0.2 μM each of primers, and 1.25 U FastStart Taq enzyme (Roche Diagnostics, Indianapolis, IN, USA). NP targets were amplified by using an initial 2-min denaturation at 95°C, followed by 40 cycles of 95°C for 30 sec, 55°C for 30 sec, and 72°C for 1 min. Some specimens produced poor PCR products, with low yields or multiple bands when we used published primer pair 1010C/OW1696R (*17*); 1 μL of PCR product from those specimens was amplified in nested PCR by using the primer pair LAS_NP_F_1/LAS_NP_R_1 and the same thermal cycling program to generate DNA fragments suitable for sequencing (Table 1). GPC targets were amplified by using 36E2 and LVS339-rev primers (*24*) and a PCR profile consisting of 2-min denaturation at 95°C, followed by 45 cycles of 95°C for 30 sec, 58°C for 30 sec, and 72°C for 1 min. L gene targets were amplified by using modified primers, LVL3359-F and LVL3754-R, based on published sequences (*28*) and a PCR program consisting of 2-min denaturation at 95°C followed by 45 cycles of 95°C for 30 sec, 53°C for 30 sec, and 72°C for 1 min. PCR amplicons were size-confirmed by electrophoresis by using 1.2% FlashGel DNA cassettes (Lonza, Walkersville, MD, USA) and purified on Zymo DNA Clean & Concentrator columns (Zymo Research, Irvine, CA, USA). All DNA sequencing was performed by Eurofins MWG Operon (Huntsville, AL, USA). The sequences were deposited into GenBank under the following accession numbers: NP sequences, KM406518–KM406556; GPC sequences, KM406590–KM406623; and L sequences, KM406557–KM406589.

### RPM-TEI Microarray Analysis

The resequencing pathogen microarray (RPM) analysis was conducted by using Tropical and Emerging Infections microarrays (RPM-TEI v. 1.0; TessArae, Potomac Falls, VA, USA). The RPM-TEI microarray enables detection of 84 biothreat agents, including all lineages of LASV (*29*). Sample preparation was conducted as previously described (*29*). Pathogen identification was performed using the “C3 Score” identification algorithm (*30*).

### Phylogenetic Analysis

We conducted the sequence alignment using the MUSCLE algorithm implemented in the MEGA 6.0 software package (*31*). In addition to partial NP, GPC, and L sequences obtained in this study, we included in the alignments all homologous sequences from these genes in samples collected in Sierra Leone (or clustering with Sierra Leone sequences) available in GenBank. Twenty-seven NP, 10 GPC, and 8 L sequences were available that meet these criteria. To root the trees, sequences from more distantly related, lineage IV isolate Z-158, which originated from Macenta district in Guinea, were used as an outgroup on the basis of the previous phylogenetic analyses (*17*). We also used MEGA 6.0 to perform statistical selection of the nucleotide substitution model for each sequence collection. We selected the Tamura 3-parameter model with discrete γ-distributed rate variation as the best-fitting model for NP and L sequence sets and the Kimura 2-parameter model with a fraction of evolutionary invariant sites for GPC sequences. The phylogenies were inferred by using the Bayesian, Markov Chain Monte Carlo method, as implemented in MrBayes v3.2.2 (*32*). The analysis was run without an assumption of a molecular clock. The resulting phylogenies were presented as 50% majority rule consensus trees in which the branches with posterior probability <0.5 were collapsed into polytomies. We manually adjusted the trees using FigTree v1.4.2 (http://tree.bio.ed.ac.uk/software/figtree).

## Results and Discussion

**Figure 1:**
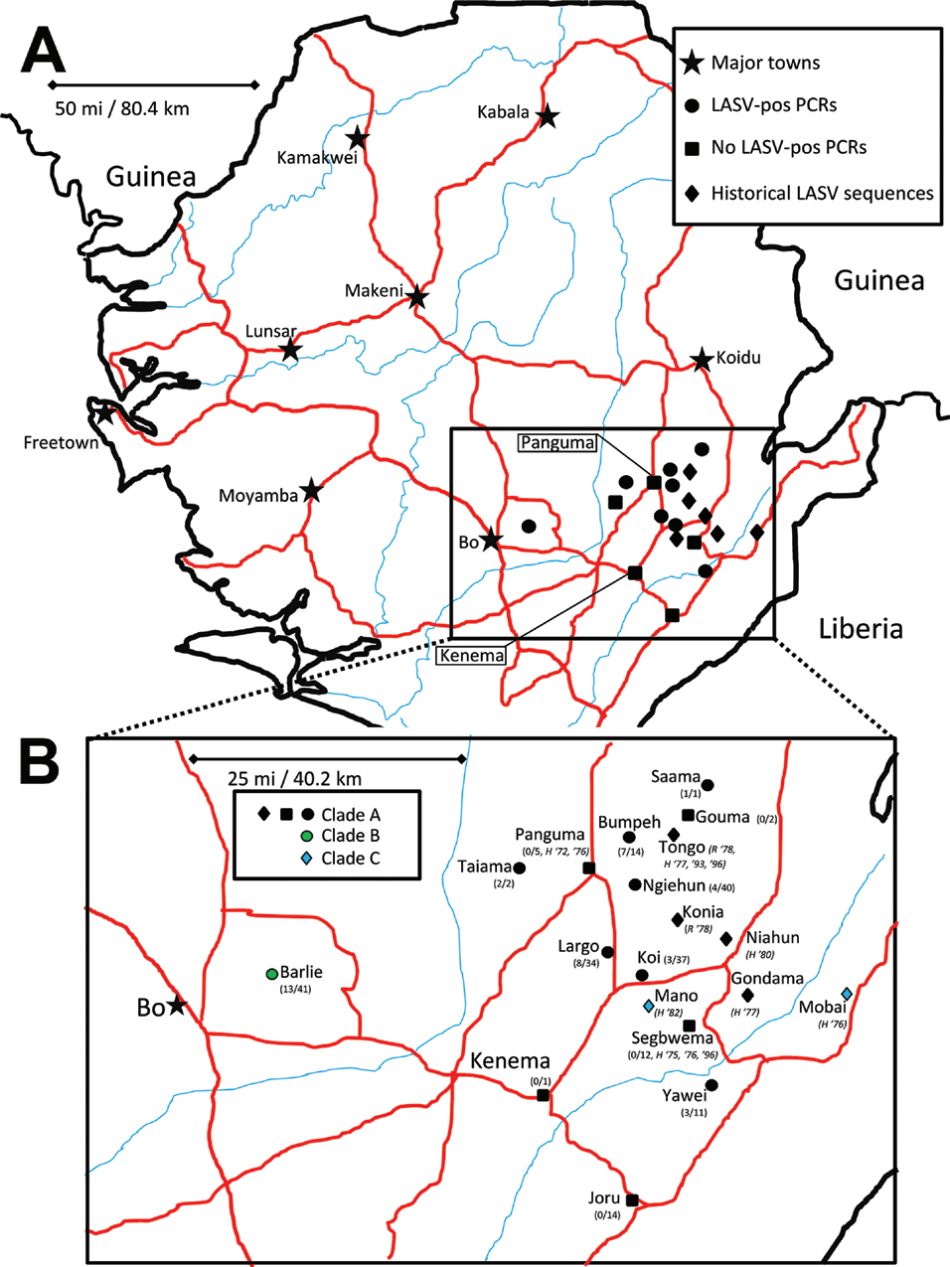
A) Locations of origin for Lassa virus (LASV) nucleic acid sequences, Sierra Leone. B) Enlarged view of region from which rodent specimens were collected. Major roads (red) and waterways (blue) are indicated. Symbols indicate major cities and towns (stars); sites in this study with rodent samples that were PCR positive for LASV (circles); sites in this study from which all samples from mulitmammate rats were PCR negative for LASV (squares); and sites from which published LASV sequences originated (diamonds). The color of the symbols in panel B indicates the clade for nucleoprotein sequence: black, clade A; green, clade B; blue, clade C. Fractions indicate, for each site included in this study, number of PCR-positive samples and total number of samples. Other designations for published sequence sites indicate type of isolate (H, human; R, rodent) and year(s) of isolation. No published information about geographic origin was available for the following strains: 807875, 331, 523, IJ531, Josiah, NL, SL06–2057, SL15, SL20, SL21, SL25, SL26, SL620.

We collected 681 small mammals during the field survey. Of these, we analyzed 214 for this study on the basis of RNA availability at the time of the study (Technical Appendix Table 2). These samples were obtained from rodents captured at 13 locations in 3 districts within the southern and eastern provinces of Sierra Leone (Figure 1; Technical Appendix Table 1). The rodents belonged to 6 genera; 199 were identified as *M. natalensis*, which is consistent with published data on the ubiquitous presence of this species in domestic environments in West Africa (*11*,*12*). The other rodents (identified to genus level only) were *Rattus* sp. (9 [4.2%] rodents); *Cricetomys* sp. (3 [1.4%] rodents); and *Mus* sp., *Praomys* sp., and *Hylomyscus* sp. (1 [0.5%] rodent each).

**Table 2:** Results of Lassa virus detection among rodent samples with >1 positive test result, Sierra Leone*

Sample	Date collected	Collection site			PCR†			RPM‡
		Village/town	District		NP	GPC	L	
LM0034	2009 Jan 27	Bumpeh	Kenema		+	+	+	Neg
LM0036	2009 Jan 27	Bumpeh	Kenema		+	+	+	NT
LM0047	2009 Jan 28	Bumpeh	Kenema		+	+	Neg	NT
LM0054	2009 Jan 28	Bumpeh	Kenema		+	+	+	NT
LM0058	2009 Jan 29	Bumpeh	Kenema		+	+	+	Neg
LM0064	2009 Jan 30	Bumpeh	Kenema		+	+	+	NT
LM0068	2009 Jan 30	Bumpeh	Kenema		+	+	Neg	Neg
LM0087	2009 Feb 3	Largo	Kenema		+	Neg	Neg	NT
LM0091	2009 Feb 3	Largo	Kenema		+	+	+	+
LM0092	2009 Feb 3	Largo	Kenema		+	+	+	NT
LM0093	2009 Feb 3	Largo	Kenema		+	+	+	NT
LM0111	2009 Feb 4	Largo	Kenema		+	+	+	NT
LM0122	2009 Feb 5	Largo	Kenema		+	+	+	NT
LM0123	2009 Feb 5	Largo	Kenema		+	+	+	NT
LM0124	2009 Feb 5	Largo	Kenema		+	+	+	NT
LM0224	2009 Feb 18	Koi	Kenema		+	+	+	NT
LM0250	2009 Feb 19	Koi	Kenema		+	+	+	NT
LM0273	2009 Feb 20	Koi	Kenema		+	+	+	NT
LM0395	2009 Jul 22	Ngiehun	Kenema		+	+	+	+
LM0396	2009 Jul 22	Ngiehun	Kenema		+	Neg	+	+
LM0434	2009 Jul 23	Ngiehun	Kenema		+	+	+	+
LM0473	2009 Jul 24	Ngiehun	Kenema		+	+	+	NT
LM0513	2009 Aug 1	Saama	Kenema		+	Neg	Neg	NT
LM0582	2009 Aug 8	Barlie	Bo		+	+	+	NT
LM0591	2009 Aug 8	Barlie	Bo		Neg	Neg	Neg	+
LM0610	2009 Aug 8	Barlie	Bo		+	+	+	+
LM0619	2009 Aug 8	Barlie	Bo		+	Neg	+	NT
LM0645	2009 Aug 8	Barlie	Bo		+	+	+	NT
LM0649	2009 Aug 8	Barlie	Bo		Neg	Neg	Neg	+
LM0657	2009 Aug 8	Barlie	Bo		+	+	+	NT
LM0660	2009 Aug 8	Barlie	Bo		+	+	+	NT
LM0661	2009 Aug 8	Barlie	Bo		+	+	+	NT
LM0676	2009 Aug 8	Barlie	Bo		+	+	+	NT
LM0677	2009 Aug 8	Barlie	Bo		+	+	+	NT
LM0678	2009 Aug 8	Barlie	Bo		+	+	+	NT
LM0680	2009 Aug 8	Barlie	Bo		+	+	+	NT
LM0714	2009 Aug 14	Yawei	Kenema		+	+	Neg	NT
LM0716	2009 Aug 14	Yawei	Kenema		+	+	+	+
LM0729	2009 Aug 15	Yawei	Kenema		+	Neg	Neg	+
Z0005	2009 Dec 17	Taiama	Kenema		+	+	+	NT
Z0007	2009 Dec 17	Taiama	Kenema		+	+	+	NT

*GPC, glycoprotein precursor; L, polymerase; Neg, negative; NP, nucleoprotein; NT, sample not tested; RPM, resequencing pathogen microarray; +, positive. †Results of PCR detection using appropriate diagnostic primers. ‡Results of RPM-TEI detection of LASV.

We screened all samples for LASV by RT-PCR using pan–Old World arenavirus (OWA) primers (*17*), which amplify a 670-nt section of NP gene. Because of poor results of NP amplification using OWA primers (inefficient amplification, multiple bands), we modified the screening protocol to include a second nested PCR using primers internal to the OWA amplification product, designed to amplify 650-nt segment of NP sequence and be more specific for lineage IV NP gene sequences. We obtained sequencing-quality NP amplicons from 39 samples using this protocol. For all of these NP-positive samples, we attempted to obtain RT-PCR amplicons for fragments of GPC and L genes using previously published or modified primers (*17*,*24*,*28*). The GPC and L amplifications were successful for most NP-positive samples and failed only in 5 and 6 samples, respectively (Table 2).

In addition, we screened a randomly selected subset of 51 samples (representing 8 collection sites and 4 rodent genera; Technical Appendix Table 2) for nucleic acids of 84 different pathogens using RPM-TEI v. 1.0. Of the 51 samples tested using RPM-TEI, 9 were positive for LASV, and no other pathogens were detected in the analyzed samples. Although the percentage of positive samples was similar to that from RT-PCR results, the RPM-TEI failed to detect LASV in 3 samples that were positive for the NP gene by 2-step RT-PCR (LM34, LM58, and LM68). All these samples originated from the same village (Bumpeh), and no sample from this site was RPM-TEI positive, which suggests that detection failures might have been caused by inefficient amplification of the sequence variant of LASV circulating in the Bumpeh area with the RPM-TEI primers. On the other hand, RPM-TEI detected viral RNA in 2 RT-PCR–negative samples (LM591 and LM649), bringing to 41 the number of samples positive for LASV nucleic acids (Table 2).

In summary, 41 LASV-positive samples were obtained from animals captured in 8 locations: Barlie (13 samples), Largo (8 samples), Bumpeh (7 samples), Ngiehun (4 samples), Koi and Yawei (3 samples each), Taiama (2 samples), and Saama (1 sample) (Figure 1). No LASV RNA was detected in samples collected from Gouma, Joru, Kenema, Panguma, or Segbwema. Lack of LASV detection in Kenema, Panguma, and Segbwema, areas well known to have regular LASV transmission, might be due to the small number of traps used. The town of Joru was extensively trapped, and no LASV was found. This finding is not surprising because Joru is south of the area where LASV is usually found.

All positive samples came from multimammate rats, which is considered the sole vector species for LASV (*13*). The results of LASV detection using several different RT-PCR strategies and a broad-range resequencing microarray (RPM-TEI v. 1.0) showed that none of the techniques applied alone detected viral RNA in all positive samples. This result underscores the difficulty of developing a truly universal diagnostic assay for this highly variable virus, even in the case of closely related strains belonging to lineage IV.

The analysis of the new sequences of LASV strains circulating in rodents in Sierra Leone indicated that the viral genome diversity is higher than previously estimated (*17*). For all available Sierra Leone sequences (including this study) the mean difference calculated for partial NP, GPC, and L sequences was 7.01% nt, 8.92% nt, and 9.83% nt, respectively, and 2.82% aa, 4.06% aa, and 0.71% aa, respectively (Table 3). These differences are higher than the reported 4.6% nt and 1.7% aa differences based on partial NP sequences in a study with fewer isolates (*17*). The L gene fragment seemed to vary the most at the nucleotide level, followed by GPC and NP, which is consistent with previous observations (*33*). However, at the amino acid level, the GPC gene varied most, followed by the NP and L genes. The high conservation of the protein sequence of L gene fragment analyzed in this study (0.71% mean difference) seemed have resulted from selection of a highly conserved part of L gene (located in RNA polymerase domain) when the diagnostic assay was designed (*28*).

**Table 3:** Estimates of average evolutionary divergence of NP, GPC, and L gene fragments for Lassa virus strains, Sierra Leone*

Gene, grouping	Difference†	
	Nucleotide	Amino acid
NP		
Overall	7.01	2.82
Clade A	5.03	2.06
Clade B	0.62	0.77
Clade C	6.44	2.42
GPC		
Overall	8.92	4.06
Clade A	6.26	2.60
Clade B	0.68	0.49
Clade D	7.49	3.29
L		
Overall	9.83	0.71
Clade A	6.59	0.58
Clade B	0.89	0.00
Clade D	0.88	0.00

*GPC, glycoprotein precursor; L, polymerase; NP, nucleoprotein. †The numbers of nucleotide and amino acid differences per site from averaging over all sequence pairs or all sequence pairs within a clade multiplied by 100 are shown. All positions containing gaps and missing data were eliminated. Values for clade E defined for GPC and L sequences were not calculated because clade E contained only 1 sequence.

**Figure 2:**
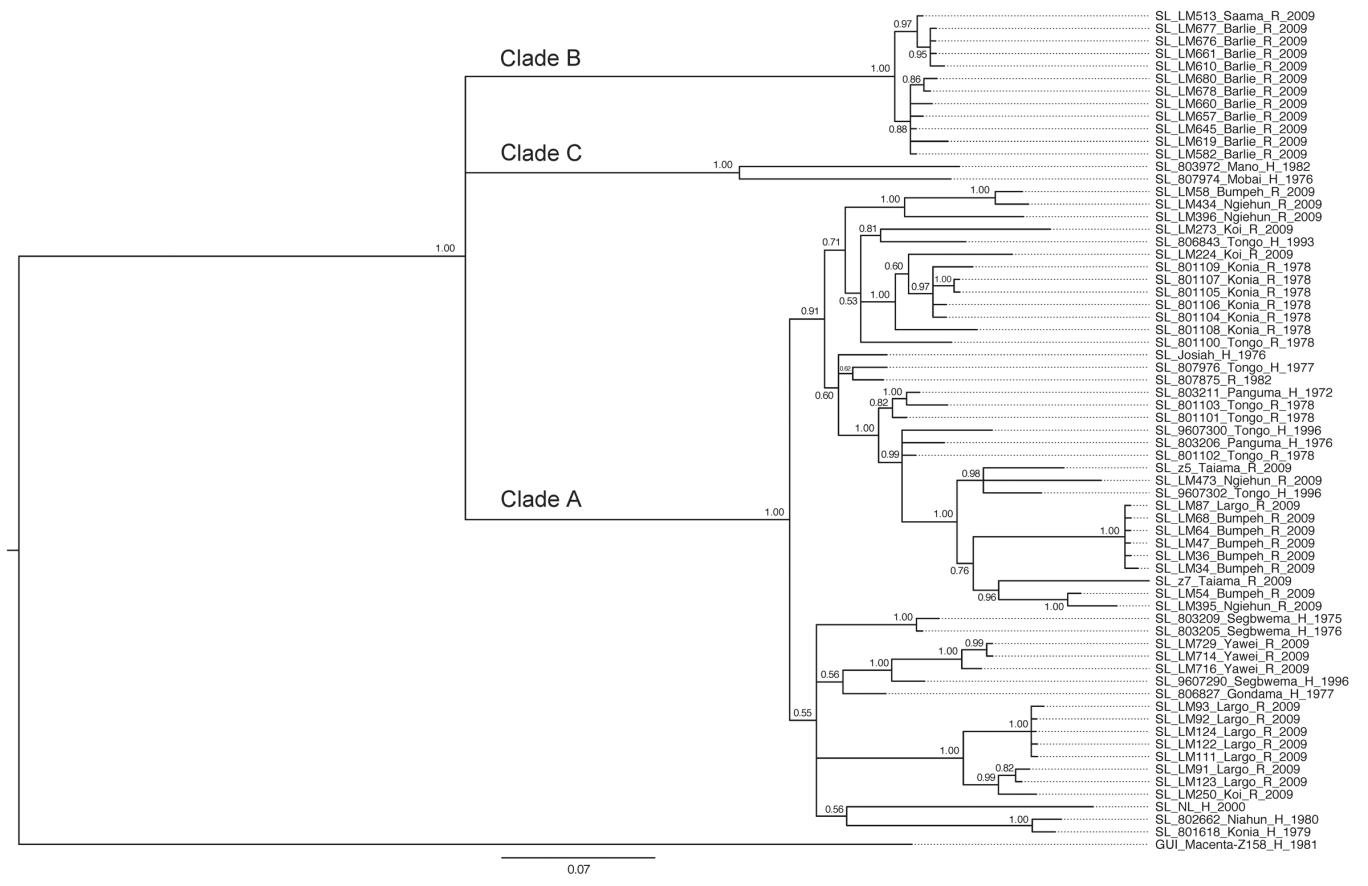
Phylogenetic analysis of Lassa virus isolates from Sierra Leone based on partial nucleoprotein (NP) gene sequences. The homologous NP fragments of 621 nt were aligned. The isolate Z-158, which originated from Macenta district in Guinea, was used as outgroup based on the previous phylogenetic analyses to root the tree. The 50% majority rule consensus tree was estimated by using Bayesian Inference method implemented in MrBayes software (*32*) using the Tamura 3-parameter substitution model with discrete γ-distributed rate variation. The strain labels contain information on the country of origin (SL, Sierra Leone; GUI, Guinea), strain designation, village or town of origin, type of isolate (H, human; R, rodent), and year of isolation. The numbers next to the branches indicate the posterior probability of particular clades. The clades as defined in this study (clades A, B, and C) are also indicated next to the appropriate branches. Scale bar indicates substitutions per site.

The analysis of phylogenetic trees constructed by using all available partial sequences of NP, GPC, and L genes from Sierra Leone confirmed previous findings that the strains circulating in this country belong to lineage IV and are closely related to each other (*17*,*19*). The topology of the largest NP-based tree (Figure 2) strongly supports the hypothesis that the isolates from Sierra Leone belong to at least 3 distinct major clades (posterior probability 1.00 in all cases): the first clade (A), including a large cluster of strains originating from a group of villages to the north and east of Kenema in the Eastern Province (Bumpeh, Gondama, Koi, Konia, Largo, Ngiehun, Panguma, Segbwema, Taiama, Tongo, and Yawei; Figure 1); the second clade (B), including several strains isolated from rodents captured in Barlie (located a few kilometers southeast of Bo) and 1 isolate from Saama (located northeast of Kenema); and the third clade (C) represented by just 2 older human isolates from Mano and Mobai.

**Figure 3:**
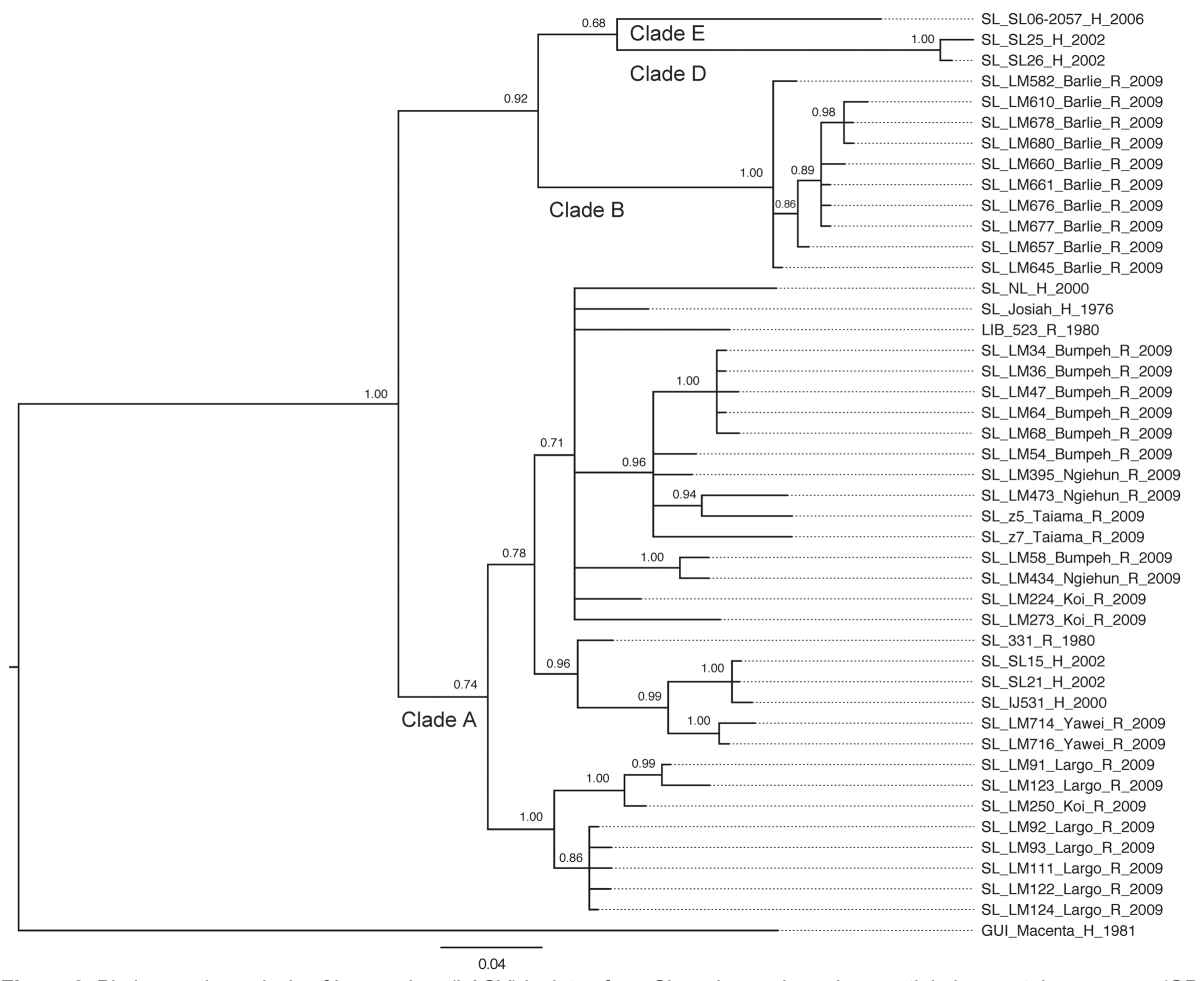
Phylogenetic analysis of Lassa virus (LASV) isolates from Sierra Leone based on partial glycoprotein precursor (GPC) gene sequences. The homologous GPC fragments of 284 nt were aligned. The isolate Z-158, which originated from Macenta district in Guinea were used as outgroup based on the previous phylogenetic analyses to root the tree. The 50% majority rule consensus tree was estimated by using Bayesian Inference method implemented in MrBayes software (*32*) using the Kimura 2-parameter substitution model with a fraction of evolutionary invariant sites. The strain labels contain information on the country of origin (SL, Sierra Leone; GUI, Guinea; LIB, Liberia), strain designation, village or town of origin, type of isolate (H, human; R, rodent), and year of isolation. The numbers next to the branches indicate the posterior probability of particular clades. The clades as defined in this study (clades A, B, D, and E) are also indicated next to the appropriate branches. Scale bar indicates substitutions per site.

**Figure 4:**
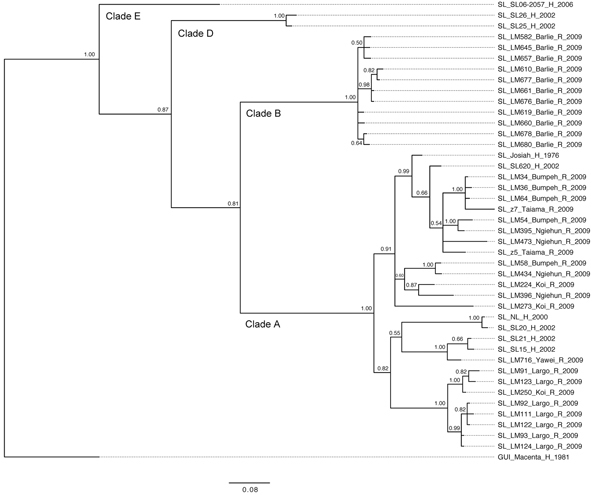
Phylogenetic analysis of Lassa virus (LASV) isolates from Sierra Leone based on partial polymerase (L) gene sequences. The homologous L fragments of 373 nt were aligned. The isolate Z-158, which originated from Macenta district in Guinea, was used as outgroup based on the previous phylogenetic analyses to root the tree. The 50% majority rule consensus tree was estimated by using Bayesian Inference method implemented in MrBayes software (*32*) using the Tamura 3-parameter substitution model with and a fraction of evolutionary invariant sites. The strain labels contain information on the country of origin (SL, Sierra Leone; GUI, Guinea), strain designation, village or town of origin, type of isolate (H, human; R, rodent), and year of isolation. The numbers next to the branches indicate the posterior probability of particular clades. The clades as defined in this study (clades A, B, D, and E) are also indicated next to the appropriate branches. Scale bar indicates substitutions per site.

Phylogenetic trees based on GPC and L sequences (Figures 3, 4) had similar topology and supported existence of clades A (with posterior probabilities 0.74 and 1.00, respectively) and B (with posterior probabilities 1.00 for both trees). However, the clade C was not present because the sequences for GPC and L gene fragments were not available for the strains forming this cluster in the NP-based tree. In addition to clades A and B, GPC- and L-based trees suggested existence of 2 additional and distinct clades. Clade D was represented by 2 sequences from human isolates SL25 and SL26, which formed a separate cluster (posterior probability 1.00 for both trees), and clade E represented by sequences obtained from a single strain isolated in 2006 (SL06–2057). These clades are defined by a very small number of sequences, and the GPC- and L-based trees disagree on the order of their separation from other clades. In addition, no data have been published on geographic origin of clade D and E samples. More data are needed (including corresponding NP sequences) to establish the existence and position of clades D and E with more certainty.

All of the trees indicate a high degree of geographic clustering of the strains. This kind of clustering has been reported previously over large geographic distances and is believed to have resulted from limited dispersal and migration of the host species (*17*,*19*). Results of this study show that this phenomenon also can be observed over relatively short distances. Isolates originating from multimammate rat specimens obtained in a particular location tended to cluster, and conversely sequences present in specific branches of the trees in many cases originated from a single location or few locations not far from each other. This kind of clustering could be observed especially well in samples from Barlie, Largo, Bumpeh, Konia, and Yawei (Figures 2–4).

In addition to the general pattern of geographic clustering, in several cases single isolates clustered with strains from different locations. For example, the sequence from a single sample from Saama (LM513) was closely related to that of strains from Barlie (based on NP sequence analysis). In another example, 1 GPC sequence originating from Liberia (523) clustered with Sierra Lone clade A sequences. In some cases (e.g., Saama sample LM513), such unusual clustering patterns may be explained by cross contamination or mislabeling of the samples. They also might result from relative proximity of all sampling sites and inadvertent anthropogenic transfer of rodents. Massive population movements that occurred in Sierra Leone during the 1991–2002 civil war could contribute to the process of mixing multimammate rat subpopulations carrying different LASV strains (*34*).

The geographic location of human cases at such a fine spatial scale can be problematic because humans can move large distances after exposure before disease is detected. For the human isolates, the clustering inconsistent with geographic location might have resulted from recording of the hospital location or patient’s current location as strain’s origin instead of the actual location of rodent–human transmission. For example, the NP-based phylogenetic tree indicates that human isolates from Segbwema and Gondama (obtained in 1996 and 1977, respectively) most likely originated from the Yawei village area because they cluster closely. A few other human isolates (SL15, SL20, and SL21) for which no location information is available also clustered with Yawei isolates on the basis of GPC and L sequences, suggesting their origin in the same area. These sequences were obtained in 2002 from United Nations peacekeepers stationed in this part of Sierra Leone (*28*,*35*).

Recent epidemiologic data show that LF was detected in 10 of 13 districts in Sierra Leone, which suggests that the infection is much more common that previously recognized (*36*). Phylogenetic analysis of the sequences revealed that strains circulating in districts to the west of the traditional hyperendemic area from which most sequence information is available differ significantly (clade B), which suggests that these could be distinct LASV strains that circulated in local multimammate rat populations for a long time since diverging from a common ancestor and are unlikely to have resulted from recent expansion of this rodent to new areas, as was recently suggested to explain emergence of cases from districts in which LF was not previously reported (*36*). Furthermore, the presence of LASV in Barlie with such high prevalence was surprising because this area historically has had few reports of LASV until 2 human LF cases reported in 2009 (L.M. Moses, unpub. data). The lack of reported LF cases from this area leads to speculation that clade B may be a less pathogenic form of LASV, and transmission to humans might have occurred previously but went unrecognized because of milder, nonhemorrhagic symptoms. In fact, the idea of broader area of LASV endemicity in Sierra Leone is consistent with results of serosurveys conducted during the 1980s by McCormick, who found seroprevalence levels ranging from 8% in southern coastal areas to 15% in villages in Northern Province (*6*).

Molecular characterization of isolates from a wider geographic area of the country is needed to fully understand the diversity of the LASV strains in Sierra Leone and its impact on disease distribution and risk. Such information would be useful for developing efficient viral detection technologies, for example, enabling design of PCR primers and antibodies specific for a broad range of LASV types. These diagnostic tests are extremely relevant to disease surveillance and monitoring and evaluation of interventions to prevent primary LASV infection in humans. More extensive information about sequence diversity affecting the antigenicity of the virus or the function of its RNA-dependent RNA polymerase may help in the development of vaccines and antiviral drugs. It will also lead to deeper understanding of the biology and pathogenesis of LASV.

Technical Appendix Rodent trapping procedures; coordinates of sampling locations; and list of all analyzed samples.
