# Supplementary material for: Sequence Variability and Geographic Distribution of Lassa Virus, Sierra Leone
Source: Emerg Infect Dis. 2015 Apr;21(4):609–18. doi: 10.3201/eid2104.141469 (PMC4378485; doi:10.3201/eid2104.141469)
Supplement: Technical Appendix — Rodent trapping procedures; coordinates of sampling locations; and list of all analyzed samples. [file 14-1469-Techapp-s1.pdf]

Article DOI: http://dx.doi.org/10.3201/eid2104.141469

Sequence Variability and Geographic Distribution of Lassa Virus, Sierra Leone

Technical Appendix

Rodent Trapping Procedures

Villages were selected if there was a laboratory-confirmed Lassa fever (LF) case originating from the village within the 6 months before the study began. Trapping using Sherman live-capture traps (H.B. Sherman Traps, Tallahassee, FL, USA) baited with oats, ground nut paste, palm oil, and dried fish was conducted in houses for 4 consecutive nights.

In 8 of the villages (Barlie, Bumpeh, Gouma, Joru, Koi, Ngiehun, Saama, Yawei), Sherman traps were placed inside all houses willing to participate in the study. Willingness to trap in houses was >95% in all villages. Two traps were placed in each room. Traps were not set outside houses because part of the aim of the original study was to measure house infestation of rodents.

In 2 of the villages (Kenema, Taiama), trapping was conducted as part of 2 case investigations for LF. Traps were set in the LF patient's house and in the adjacent houses if they were willing to participate. Two Sherman traps were placed in each room of the houses trapped.

In 3 of the villages (Largo, Panguma, Segbwema), trapping was done on 2 streets for training purposes. Streets were selected because a contact was residing on the street. Sherman traps were placed in all houses willing to participate in the study. Two Sherman traps were placed in each room of the houses trapped.

In 1 village (Bumpeh), 3 *Cricetomys* were collected from trappers that used locally made wooden live-capture traps baited with cassava root and were placed overnight in the bush surrounding the village. These traps target this large rodent. Specimen processing was done as described for other small mammals collected.

All traps (Sherman and locally made) were set and baited in the evening and checked just after sunrise.

Technical Appendix Table 1. Coordinates of sampling locations

Town	GPS coordinates	Positive samples/total samples
Barlie	8.02, -11.58	13/41
Largo	8.05, -11.11	8/34
Bumpeh	8.21, -11.09	7/14
Ngiehun	8.18, -11.08	4/40
Koi	8.04, -11.06	3/37
Yawei	7.82, -10.95	3/11
Taiama	8.17, -11.23	2/2
Saama	8.28, -11.01	1/1
Joru	7.69, -11.06	0/14
Segbwema	8.00, -10.95	0/12
Panguma	8.19, -11.13	0/5
Gouma	8.26, -11.02	0/2
Kenema	8.15, -11.25	0/1

Technical Appendix Table 2. All analyzed samples

	Date	Collection Site				PCR			
Sample	Collected	Village/Town	District	Rodent genus	Rodent species	NP	GPC	L	RPM
LM0018	01/15/09	Kenema	Kenema	*Mus*	undetermined	neg	neg	neg	NT
LM0022	01/20/09	Panguma	Kenema	*Mastomys*	*natalensis*	neg	neg	neg	neg
LM0023	01/20/09	Panguma	Kenema	*Mastomys*	*natalensis*	neg	neg	neg	neg
LM0024	01/20/09	Panguma	Kenema	*Mastomys*	*natalensis*	neg	neg	neg	NT
LM0026	01/20/09	Panguma	Kenema	*Mastomys*	*natalensis*	neg	neg	neg	NT
LM0028	01/21/09	Panguma	Kenema	*Rattus*	undetermined	neg	neg	neg	NT
LM0034	01/27/09	Bumpeh	Kenema	*Mastomys*	*natalensis*	pos	pos	pos	neg
LM0036	01/27/09	Bumpeh	Kenema	*Mastomys*	*natalensis*	pos	pos	pos	NT
LM0037	01/27/09	Bumpeh	Kenema	*Rattus*	undetermined	neg	neg	neg	NT
LM0047	01/28/09	Bumpeh	Kenema	*Mastomys*	*natalensis*	pos	pos	neg	NT
LM0054	01/28/09	Bumpeh	Kenema	*Mastomys*	*natalensis*	pos	pos	pos	NT
LM0058	01/29/09	Bumpeh	Kenema	*Mastomys*	*natalensis*	pos	pos	pos	neg
LM0059	01/29/09	Bumpeh	Kenema	*Mastomys*	*natalensis*	neg	neg	neg	neg
LM0064	01/30/09	Bumpeh	Kenema	*Mastomys*	*natalensis*	pos	pos	pos	NT
LM0067	01/30/09	Bumpeh	Kenema	*Mastomys*	*natalensis*	neg	neg	neg	NT
LM0068	01/30/09	Bumpeh	Kenema	*Mastomys*	*natalensis*	pos	pos	neg	neg
LM0069	01/30/09	Bumpeh	Kenema	*Mastomys*	*natalensis*	neg	neg	neg	NT
LM0070	01/30/09	Bumpeh	Kenema	*Mastomys*	*natalensis*	neg	neg	neg	NT
LM0077	01/30/09	Bumpeh	Kenema	*Cricetomys*	undetermined	neg	neg	neg	neg
LM0078	01/30/09	Bumpeh	Kenema	*Cricetomys*	undetermined	neg	neg	neg	neg
LM0079	02/03/09	Largo	Kenema	*Mastomys*	*natalensis*	neg	neg	neg	NT
LM0080	02/03/09	Largo	Kenema	*Mastomys*	*natalensis*	neg	neg	neg	neg
LM0081	02/03/09	Largo	Kenema	*Mastomys*	*natalensis*	neg	neg	neg	NT
LM0082	02/03/09	Largo	Kenema	*Mastomys*	*natalensis*	neg	neg	neg	NT
LM0083	02/03/09	Largo	Kenema	*Mastomys*	*natalensis*	neg	neg	neg	NT
LM0084	02/03/09	Largo	Kenema	*Mastomys*	*natalensis*	neg	neg	neg	neg
LM0085	02/03/09	Largo	Kenema	*Mastomys*	*natalensis*	neg	neg	neg	NT
LM0086	02/03/09	Largo	Kenema	*Mastomys*	*natalensis*	neg	neg	neg	NT
LM0087	02/03/09	Largo	Kenema	*Mastomys*	*natalensis*	pos	neg	neg	NT
LM0088	02/03/09	Largo	Kenema	*Mastomys*	*natalensis*	neg	neg	neg	NT
LM0090	02/03/09	Largo	Kenema	*Mastomys*	*natalensis*	neg	neg	neg	NT
LM0091	02/03/09	Largo	Kenema	*Mastomys*	*natalensis*	pos	pos	pos	pos
LM0092	02/03/09	Largo	Kenema	*Mastomys*	*natalensis*	pos	pos	pos	NT
LM0093	02/03/09	Largo	Kenema	*Mastomys*	*natalensis*	pos	pos	pos	NT
LM0094	02/03/09	Largo	Kenema	*Mastomys*	*natalensis*	neg	neg	neg	NT
LM0097	02/03/09	Largo	Kenema	*Mastomys*	*natalensis*	neg	neg	neg	NT
LM0100	02/03/09	Largo	Kenema	*Mastomys*	*natalensis*	neg	neg	neg	neg
LM0101	02/03/09	Largo	Kenema	*Mastomys*	*natalensis*	neg	neg	neg	NT
LM0104	02/04/09	Largo	Kenema	*Mastomys*	*natalensis*	neg	neg	neg	NT
LM0105	02/04/09	Largo	Kenema	*Mastomys*	*natalensis*	neg	neg	neg	NT
LM0107	02/04/09	Largo	Kenema	*Mastomys*	*natalensis*	neg	neg	neg	NT
LM0108	02/04/09	Largo	Kenema	*Mastomys*	*natalensis*	neg	neg	neg	NT
LM0109	02/04/09	Largo	Kenema	*Mastomys*	*natalensis*	neg	neg	neg	NT
LM0111	02/04/09	Largo	Kenema	*Mastomys*	*natalensis*	pos	pos	pos	NT
LM0114	02/04/09	Largo	Kenema	*Mastomys*	*natalensis*	neg	neg	neg	NT
LM0115	02/05/09	Largo	Kenema	*Mastomys*	*natalensis*	neg	neg	neg	NT
LM0116	02/05/09	Largo	Kenema	*Mastomys*	*natalensis*	neg	neg	neg	NT
LM0122	02/05/09	Largo	Kenema	*Mastomys*	*natalensis*	pos	pos	pos	NT
LM0123	02/05/09	Largo	Kenema	*Mastomys*	*natalensis*	pos	pos	pos	NT

	Date	Collection Site				PCR			
Sample	Collected	Village/Town	District	Rodent genus	Rodent species	NP	GPC	L	RPM
LM0124	02/05/09	Largo	Kenema	*Mastomys*	*natalensis*	pos	pos	pos	NT
LM0125	02/05/09	Largo	Kenema	*Cricetomys*	undetermined	neg	neg	neg	neg
LM0126	02/06/09	Largo	Kenema	*Mastomys*	*natalensis*	neg	neg	neg	NT
LM0131	02/06/09	Largo	Kenema	*Mastomys*	*natalensis*	neg	neg	neg	NT
LM0135	02/06/09	Largo	Kenema	*Mastomys*	*natalensis*	neg	neg	neg	NT
LM0137	02/10/09	Segbwema	Kailahun	*Mastomys*	*natalensis*	neg	neg	neg	NT
LM0139	02/10/09	Segbwema	Kailahun	*Mastomys*	*natalensis*	neg	neg	neg	NT
LM0141	02/10/09	Segbwema	Kailahun	*Mastomys*	*natalensis*	neg	neg	neg	NT
LM0154	02/11/09	Segbwema	Kailahun	*Mastomys*	*natalensis*	neg	neg	neg	neg
LM0159	02/12/09	Segbwema	Kailahun	*Mastomys*	*natalensis*	neg	neg	neg	NT
LM0160	02/12/09	Segbwema	Kailahun	*Rattus*	undetermined	neg	neg	neg	NT
LM0162	02/13/09	Segbwema	Kailahun	*Mastomys*	*natalensis*	neg	neg	neg	NT
LM0163	02/13/09	Segbwema	Kailahun	*Mastomys*	*natalensis*	neg	neg	neg	NT
LM0164	02/13/09	Segbwema	Kailahun	*Mastomys*	*natalensis*	neg	neg	neg	NT
LM0167	02/13/09	Segbwema	Kailahun	*Mastomys*	*natalensis*	neg	neg	neg	NT
LM0169	02/13/09	Segbwema	Kailahun	*Mastomys*	*natalensis*	neg	neg	neg	NT
LM0171	02/13/09	Segbwema	Kailahun	*Mastomys*	*natalensis*	neg	neg	neg	NT
LM0181	02/17/09	Koi	Kenema	*Mastomys*	*natalensis*	neg	neg	neg	NT
LM0190	02/17/09	Koi	Kenema	*Mastomys*	*natalensis*	neg	neg	neg	NT
LM0192	02/17/09	Koi	Kenema	*Mastomys*	*natalensis*	neg	neg	neg	NT
LM0195	02/17/09	Koi	Kenema	*Mastomys*	*natalensis*	neg	neg	neg	NT
LM0203	02/17/09	Koi	Kenema	*Mastomys*	*natalensis*	neg	neg	neg	NT
LM0205	02/17/09	Koi	Kenema	*Mastomys*	*natalensis*	neg	neg	neg	NT
LM0206	02/17/09	Koi	Kenema	*Rattus*	undetermined	neg	neg	neg	NT
LM0210	02/17/09	Koi	Kenema	*Mastomys*	*natalensis*	neg	neg	neg	NT
LM0211	02/17/09	Koi	Kenema	*Mastomys*	*natalensis*	neg	neg	neg	NT
LM0214	02/18/09	Koi	Kenema	*Mastomys*	*natalensis*	neg	neg	neg	NT
LM0216	02/18/09	Koi	Kenema	*Mastomys*	*natalensis*	neg	neg	neg	NT
LM0222	02/18/09	Koi	Kenema	*Mastomys*	*natalensis*	neg	neg	neg	NT
LM0224	02/18/09	Koi	Kenema	*Mastomys*	*natalensis*	pos	pos	pos	NT
LM0226	02/18/09	Koi	Kenema	*Mastomys*	*natalensis*	neg	neg	neg	NT
LM0227	02/18/09	Koi	Kenema	*Mastomys*	*natalensis*	neg	neg	neg	NT
LM0228	02/18/09	Koi	Kenema	*Mastomys*	*natalensis*	neg	neg	neg	NT
LM0229	02/18/09	Koi	Kenema	*Mastomys*	*natalensis*	neg	neg	neg	NT
LM0230	02/18/09	Koi	Kenema	*Mastomys*	*natalensis*	neg	neg	neg	NT
LM0231	02/18/09	Koi	Kenema	*Mastomys*	*natalensis*	neg	neg	neg	NT
LM0242	02/19/09	Koi	Kenema	*Mastomys*	*natalensis*	neg	neg	neg	NT
LM0243	02/19/09	Koi	Kenema	*Mastomys*	*natalensis*	neg	neg	neg	NT
LM0247	02/19/09	Koi	Kenema	*Mastomys*	*natalensis*	neg	neg	neg	NT
LM0249	02/19/09	Koi	Kenema	*Mastomys*	*natalensis*	neg	neg	neg	NT
LM0250	02/19/09	Koi	Kenema	*Mastomys*	*natalensis*	pos	pos	pos	NT
LM0251	02/19/09	Koi	Kenema	*Mastomys*	*natalensis*	neg	neg	neg	NT
LM0255	02/19/09	Koi	Kenema	*Mastomys*	*natalensis*	neg	neg	neg	NT
LM0260	02/20/09	Koi	Kenema	*Mastomys*	*natalensis*	neg	neg	neg	NT
LM0262	02/20/09	Koi	Kenema	*Mastomys*	*natalensis*	neg	neg	neg	NT
LM0263	02/20/09	Koi	Kenema	*Mastomys*	*natalensis*	neg	neg	neg	NT
LM0264	02/20/09	Koi	Kenema	*Mastomys*	*natalensis*	neg	neg	neg	NT
LM0267	02/20/09	Koi	Kenema	*Mastomys*	*natalensis*	neg	neg	neg	NT
LM0268	02/20/09	Koi	Kenema	*Mastomys*	*natalensis*	neg	neg	neg	NT
LM0269	02/20/09	Koi	Kenema	*Mastomys*	*natalensis*	neg	neg	neg	NT
LM0270	02/20/09	Koi	Kenema	*Mastomys*	*natalensis*	neg	neg	neg	NT
LM0271	02/20/09	Koi	Kenema	*Mastomys*	*natalensis*	neg	neg	neg	NT
LM0272	02/20/09	Koi	Kenema	*Mastomys*	*natalensis*	neg	neg	neg	NT
LM0273	02/20/09	Koi	Kenema	*Mastomys*	*natalensis*	pos	pos	pos	NT
LM0342	07/17/09	Joru	Kenema	*Mastomys*	*natalensis*	neg	neg	neg	NT
LM0351	07/17/09	Joru	Kenema	*Mastomys*	*natalensis*	neg	neg	neg	NT
LM0353	07/17/09	Joru	Kenema	*Praomys*	undetermined	neg	neg	neg	neg
LM0354	07/17/09	Joru	Kenema	*Mastomys*	*natalensis*	neg	neg	neg	NT
LM0356	07/17/09	Joru	Kenema	*Mastomys*	*natalensis*	neg	neg	neg	NT
LM0357	07/17/09	Joru	Kenema	*Mastomys*	*natalensis*	neg	neg	neg	neg
LM0358	07/17/09	Joru	Kenema	*Mastomys*	*natalensis*	neg	neg	neg	NT
LM0359	07/17/09	Joru	Kenema	*Mastomys*	*natalensis*	neg	neg	neg	NT
LM0360	07/17/09	Joru	Kenema	*Mastomys*	*natalensis*	neg	neg	neg	NT
LM0362	07/17/09	Joru	Kenema	*Mastomys*	*natalensis*	neg	neg	neg	NT
LM0365	07/17/09	Joru	Kenema	*Rattus*	undetermined	neg	neg	neg	NT
LM0366	07/17/09	Joru	Kenema	*Hylomyscus*	undetermined	neg	neg	neg	neg
LM0369	07/18/09	Joru	Kenema	*Mastomys*	*natalensis*	neg	neg	neg	NT
LM0370	07/18/09	Joru	Kenema	*Mastomys*	*natalensis*	neg	neg	neg	neg

	Date	Collection Site				PCR			
Sample	Collected	Village/Town	District	Rodent genus	Rodent species	NP	GPC	L	RPM
LM0385	07/22/09	Ngiehun	Kenema	*Mastomys*	*natalensis*	neg	neg	neg	neg
LM0395	07/22/09	Ngiehun	Kenema	*Mastomys*	*natalensis*	pos	pos	pos	pos
LM0396	07/22/09	Ngiehun	Kenema	*Mastomys*	*natalensis*	pos	neg	pos	pos
LM0405	07/22/09	Ngiehun	Kenema	*Mastomys*	*natalensis*	neg	neg	neg	NT
LM0406	07/22/09	Ngiehun	Kenema	*Mastomys*	*natalensis*	neg	neg	neg	neg
LM0411	07/22/09	Ngiehun	Kenema	*Mastomys*	*natalensis*	neg	neg	neg	neg
LM0412	07/22/09	Ngiehun	Kenema	*Mastomys*	*natalensis*	neg	neg	neg	neg
LM0415	07/22/09	Ngiehun	Kenema	*Mastomys*	*natalensis*	neg	neg	neg	neg
LM0432	07/23/09	Ngiehun	Kenema	*Mastomys*	*natalensis*	neg	neg	neg	neg
LM0433	07/23/09	Ngiehun	Kenema	*Mastomys*	*natalensis*	neg	neg	neg	NT
LM0434	07/23/09	Ngiehun	Kenema	*Mastomys*	*natalensis*	pos	pos	pos	pos
LM0435	07/23/09	Ngiehun	Kenema	*Mastomys*	*natalensis*	neg	neg	neg	NT
LM0436	07/23/09	Ngiehun	Kenema	*Mastomys*	*natalensis*	neg	neg	neg	NT
LM0437	07/23/09	Ngiehun	Kenema	*Mastomys*	*natalensis*	neg	neg	neg	neg
LM0438	07/23/09	Ngiehun	Kenema	*Mastomys*	*natalensis*	neg	neg	neg	NT
LM0439	07/23/09	Ngiehun	Kenema	*Mastomys*	*natalensis*	neg	neg	neg	neg
LM0440	07/23/09	Ngiehun	Kenema	*Mastomys*	*natalensis*	neg	neg	neg	NT
LM0441	07/23/09	Ngiehun	Kenema	*Mastomys*	*natalensis*	neg	neg	neg	NT
LM0442	07/23/09	Ngiehun	Kenema	*Mastomys*	*natalensis*	neg	neg	neg	neg
LM0443	07/23/09	Ngiehun	Kenema	*Mastomys*	*natalensis*	neg	neg	neg	neg
LM0444	07/23/09	Ngiehun	Kenema	*Mastomys*	*natalensis*	neg	neg	neg	NT
LM0445	07/23/09	Ngiehun	Kenema	*Mastomys*	*natalensis*	neg	neg	neg	NT
LM0446	07/23/09	Ngiehun	Kenema	*Mastomys*	*natalensis*	neg	neg	neg	NT
LM0447	07/23/09	Ngiehun	Kenema	*Mastomys*	*natalensis*	neg	neg	neg	NT
LM0448	07/23/09	Ngiehun	Kenema	*Mastomys*	*natalensis*	neg	neg	neg	NT
LM0449	07/23/09	Ngiehun	Kenema	*Mastomys*	*natalensis*	neg	neg	neg	NT
LM0450	07/23/09	Ngiehun	Kenema	*Mastomys*	*natalensis*	neg	neg	neg	NT
LM0451	07/23/09	Ngiehun	Kenema	*Mastomys*	*natalensis*	neg	neg	neg	NT
LM0452	07/23/09	Ngiehun	Kenema	*Mastomys*	*natalensis*	neg	neg	neg	NT
LM0460	07/22/09	Ngiehun	Kenema	*Rattus*	undetermined	neg	neg	neg	NT
LM0468	07/23/09	Ngiehun	Kenema	*Rattus*	undetermined	neg	neg	neg	NT
LM0470	07/24/09	Ngiehun	Kenema	*Mastomys*	*natalensis*	neg	neg	neg	NT
LM0472	07/24/09	Ngiehun	Kenema	*Mastomys*	*natalensis*	neg	neg	neg	NT
LM0473	07/24/09	Ngiehun	Kenema	*Mastomys*	*natalensis*	pos	pos	pos	NT
LM0474	07/24/09	Ngiehun	Kenema	*Mastomys*	*natalensis*	neg	neg	neg	NT
LM0475	07/24/09	Ngiehun	Kenema	*Mastomys*	*natalensis*	neg	neg	neg	NT
LM0476	07/24/09	Ngiehun	Kenema	*Mastomys*	*natalensis*	neg	neg	neg	NT
LM0477	07/24/09	Ngiehun	Kenema	*Mastomys*	*natalensis*	neg	neg	neg	NT
LM0479	07/24/09	Ngiehun	Kenema	*Mastomys*	*natalensis*	neg	neg	neg	NT
LM0483	07/24/09	Ngiehun	Kenema	*Mastomys*	*natalensis*	neg	neg	neg	NT
LM0513	08/01/09	Saama	Kenema	*Mastomys*	*natalensis*	pos	neg	neg	NT
LM0569	08/08/09	Barlie	Bo	*Mastomys*	*natalensis*	neg	neg	neg	NT
LM0582	08/08/09	Barlie	Bo	*Mastomys*	*natalensis*	pos	pos	pos	NT
LM0590	08/08/09	Barlie	Bo	*Mastomys*	*natalensis*	neg	neg	neg	neg
LM0591	08/08/09	Barlie	Bo	*Mastomys*	*natalensis*	neg	neg	neg	pos
LM0597	08/08/09	Barlie	Bo	*Mastomys*	*natalensis*	neg	neg	neg	NT
LM0607	08/08/09	Barlie	Bo	*Mastomys*	*natalensis*	neg	neg	neg	NT
LM0608	08/08/09	Barlie	Bo	*Mastomys*	*natalensis*	neg	neg	neg	neg
LM0609	08/08/09	Barlie	Bo	*Mastomys*	*natalensis*	neg	neg	neg	neg
LM0610	08/08/09	Barlie	Bo	*Mastomys*	*natalensis*	pos	pos	pos	pos
LM0611	08/08/09	Barlie	Bo	*Mastomys*	*natalensis*	neg	neg	neg	neg
LM0614	08/08/09	Barlie	Bo	*Mastomys*	*natalensis*	neg	neg	neg	NT
LM0615	08/08/09	Barlie	Bo	*Mastomys*	*natalensis*	neg	neg	neg	neg
LM0616	08/08/09	Barlie	Bo	*Mastomys*	*natalensis*	neg	neg	neg	neg
LM0617	08/08/09	Barlie	Bo	*Mastomys*	*natalensis*	neg	neg	neg	neg
LM0618	08/08/09	Barlie	Bo	*Mastomys*	*natalensis*	neg	neg	neg	NT
LM0619	08/08/09	Barlie	Bo	*Mastomys*	*natalensis*	pos	neg	pos	NT
LM0620	08/08/09	Barlie	Bo	*Mastomys*	*natalensis*	neg	neg	neg	NT
LM0621	08/08/09	Barlie	Bo	*Mastomys*	*natalensis*	neg	neg	neg	NT
LM0624	08/08/09	Barlie	Bo	*Mastomys*	*natalensis*	neg	neg	neg	NT
LM0626	08/08/09	Barlie	Bo	*Mastomys*	*natalensis*	neg	neg	neg	neg
LM0627	08/08/09	Barlie	Bo	*Mastomys*	*natalensis*	neg	neg	neg	neg
LM0631	08/08/09	Barlie	Bo	*Mastomys*	*natalensis*	neg	neg	neg	neg
LM0643	08/08/09	Barlie	Bo	*Mastomys*	*natalensis*	neg	neg	neg	NT
LM0644	08/08/09	Barlie	Bo	*Mastomys*	*natalensis*	neg	neg	neg	NT
LM0645	08/08/09	Barlie	Bo	*Mastomys*	*natalensis*	pos	pos	pos	NT
LM0647	08/08/09	Barlie	Bo	*Mastomys*	*natalensis*	neg	neg	neg	neg
LM0648	08/08/09	Barlie	Bo	*Mastomys*	*natalensis*	neg	neg	neg	neg

Sample	Date Collected	Collection Site		Rodent genus	Rodent species	PCR			RPM
		Village/Town	District			NP	GPC	L	
LM0649	08/08/09	Barlie	Bo	*Mastomys*	*natalensis*	neg	neg	neg	pos
LM0650	08/08/09	Barlie	Bo	*Mastomys*	*natalensis*	neg	neg	neg	neg
LM0653	08/08/09	Barlie	Bo	*Mastomys*	*natalensis*	neg	neg	neg	NT
LM0654	08/08/09	Barlie	Bo	*Mastomys*	*natalensis*	neg	neg	neg	NT
LM0657	08/08/09	Barlie	Bo	*Mastomys*	*natalensis*	pos	pos	pos	NT
LM0660	08/08/09	Barlie	Bo	*Mastomys*	*natalensis*	pos	pos	pos	NT
LM0661	08/08/09	Barlie	Bo	*Mastomys*	*natalensis*	pos	pos	pos	NT
LM0667	08/08/09	Barlie	Bo	*Rattus*	undetermined	neg	neg	neg	NT
LM0670	08/08/09	Barlie	Bo	*Mastomys*	*natalensis*	neg	neg	neg	NT
LM0671	08/08/09	Barlie	Bo	*Mastomys*	*natalensis*	neg	neg	neg	NT
LM0676	08/08/09	Barlie	Bo	*Mastomys*	*natalensis*	pos	pos	pos	NT
LM0677	08/08/09	Barlie	Bo	*Mastomys*	*natalensis*	pos	pos	pos	NT
LM0678	08/08/09	Barlie	Bo	*Mastomys*	*natalensis*	pos	pos	pos	NT
LM0680	08/08/09	Barlie	Bo	*Mastomys*	*natalensis*	pos	pos	pos	NT
LM0692	08/12/09	Yawei	Kenema	*Mastomys*	*natalensis*	neg	neg	neg	NT
LM0699	08/13/09	Yawei	Kenema	*Rattus*	undetermined	neg	neg	neg	NT
LM0703	08/13/09	Yawei	Kenema	*Mastomys*	*natalensis*	neg	neg	neg	NT
LM0714	08/14/09	Yawei	Kenema	*Mastomys*	*natalensis*	pos	pos	neg	NT
LM0716	08/14/09	Yawei	Kenema	*Mastomys*	*natalensis*	pos	pos	pos	pos
LM0717	08/14/09	Yawei	Kenema	*Mastomys*	*natalensis*	neg	neg	neg	neg
LM0719	08/14/09	Yawei	Kenema	*Mastomys*	*natalensis*	neg	neg	neg	NT
LM0722	08/14/09	Yawei	Kenema	*Mastomys*	*natalensis*	neg	neg	neg	NT
LM0725	08/15/09	Yawei	Kenema	*Mastomys*	*natalensis*	neg	neg	neg	NT
LM0729	08/15/09	Yawei	Kenema	*Mastomys*	*natalensis*	pos	neg	neg	pos
LM0731	08/15/09	Yawei	Kenema	*Mastomys*	*natalensis*	neg	neg	neg	neg
LM0747	08/20/09	Gouma	Kenema	*Mastomys*	*natalensis*	neg	neg	neg	NT
LM0760	08/21/09	Gouma	Kenema	*Mastomys*	*natalensis*	neg	neg	neg	NT
Z0005	12/17/09	Taiama	Kenema	*Mastomys*	*natalensis*	pos	pos	pos	NT
Z0007	12/17/09	Taiama	Kenema	*Mastomys*	*natalensis*	pos	pos	pos	NT

neg – negative; NT, not tested; pos, positive.
